# Multi‐omics integration analysis identifies INPP4B as a T‐cell‐specific activation suppressor

**DOI:** 10.1002/ctm2.70430

**Published:** 2025-08-03

**Authors:** Ting Peng, Qing Fang, Zihao Zhao, Yingjun Chang, Xiangyu Zhao, Cheng Li

**Affiliations:** ^1^ School of Life Sciences Peking University Beijing China; ^2^ State Key Laboratory of Molecular Oncology, Beijing Key Laboratory of Carcinogenesis and Translational Research, Department of Molecular Oncology Peking University Cancer Hospital & Institute Beijing China; ^3^ National Clinical Research Center for Hematologic Disease, Peking University Institute of Hematology, Beijing Key Laboratory of Hematopoietic Stem Cell Transplantation Peking University People's Hospital Beijing China; ^4^ Center for Statistical Science Peking University Beijing China

**Keywords:** INPP4B, multi‐omics, T‐cell activation

## Abstract

**Background:**

Naïve T cells are maintained in a quiescent state prior to activation. As inappropriate T‐cell activation can lead to impaired immune tolerance and autoimmune diseases, the transition from quiescence to activation must be under strict regulation. Despite its importance, the mechanisms underlying the maintenance of the quiescent state remain incompletely understood.

**Methods and Results:**

Through multi‐omics integration analysis, we reveal that *INPP4B*, a phosphatase of the phosphoinositide 3‐kinase pathway, is highly expressed specifically in T cells and is involved in suppressing T‐cell activation and maintaining quiescence. Our findings uncover that *INPP4B* forms a T‐cell‐specific chromatin interaction domain and exhibits high expression levels in quiescent T cells. Upon T‐cell activation, both the chromatin interaction and expression levels of *INPP4B* decrease. Functional studies further confirm that INPP4B suppresses T‐cell activation and effector functions. Additionally, we observe increased expression level of *INPP4B* in exhausted T cells within the tumour microenvironment.

**Conclusion:**

These results highlight the importance of maintaining optimal levels of *INPP4B* for T‐cell function. Our findings suggest that INPP4B could be a potential target for enhancing the efficacy of T‐cell‐mediated immune responses against tumours.

**Key points:**

1. A comprehensive multi‐omics analysis characterizes the expression patterns of INPP4B across immune populations.
2. INPP4B exhibits a T‐cell‐specific expression domain and functions as a T cell activation suppressor.
3. INPP4B is significantly upregulated in exhausted T cells within the tumour microenvironment.

## Graphical abstract

- A comprehensive multi‐omics characterisation of INPP4B across immune cell populations.
- Discovery of a T‐cell‐specific expression and interaction domain of INPP4B.
- INPP4B functions as a T‐cell‐specific activation suppressor.
- INPP4B is upregulated in exhausted T cells within the tumour microenvironment.

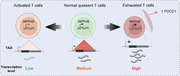

## 1 INTRODUCTION

Naïve T cells are actively maintained in a quiescent state, characterised by arrest in the G0 stage of the cell cycle, exhibiting low levels of transcriptional, translational and metabolic activities.^1^ T‐cell quiescence is a state of reversible growth arrest in which cells remain responsive to activation signals and resistant to apoptosis. It must be actively maintained by the action of a series of molecules including transcription factors (TFs) and cell‐cycle regulators.^2^ The quiescence restricts the unnecessary energy consumption by the large T lymphocyte repertoires. Moreover, maintaining T cells in a quiescent state helps prevent potential genetic damage resulting from DNA replication, thus reducing the risk of leukaemia and lymphoma development.^3^, ^4^ Loss of T‐cell quiescence can lead to impaired immune tolerance and autoimmune diseases, such as type 1 diabetes and multiple sclerosis (MS),^5^ underscoring the importance of tightly regulating T‐cell quiescence. In addition, disruption of T‐cell quiescence is associated with T‐cell acute lymphoblastic leukaemia (T‐ALL), an aggressive malignancy characterised by the rapid proliferation of immature T cells.^6^ Previous studies have elucidated that loss of T‐cell quiescence due to mutations leads to the acquisition of an aberrant developmental program, causing the impairment of T cells renewal capabilities and leading to the development of T‐ALL.^7^, ^8^

The transition of T cells from quiescence to activation is controlled by the engagement of T‐cell receptor (TCR) and an array of co‐stimulatory molecules, such as CD28, which encounter binding partners during antigen presentation.^9^ T‐cell activation is associated with a shift in signalling and transcriptional programs.^1^, ^10^, ^11^ Phosphoinositide 3‐kinase (PI3K) signalling activation is a seminal event in this process, as it initiates a cascade of pathways involved in regulating cellular function, including protein kinase B (AKT), mechanistic target of rapamycin (mTOR) complex 1 (mTORC1) and mTORC2. PI3K signalling plays a pivotal role in regulating multiple aspects of T‐cell biology, including activation, proliferation, differentiation and effector responses. Precise control of PI3K pathway activity is particularly critical for maintaining the balance between T‐cell activation and quiescence, and its dysregulation has been implicated in T‐cell exhaustion and T‐ALL.^12^, ^13^, ^14^

To prevent such pathological outcomes, the PI3K pathway is tightly regulated by a network of lipid phosphatases, including phosphatase and tensin homolog (PTEN) and SH2‐domain containing inositol polyphosphate 5‐phosphatase (SHIP), which act to terminate or modulate PI3K signalling through specific dephosphorylation of PIP_3_ and PI(3,4)P_2_. However, beyond these well‐studied regulators, the contribution of downstream phosphatases such as INPP4B—which specifically converts PI(3,4)P_2_ to PI(3)P—remains largely unexplored in T cells. Given the central role of PI(3,4)P_2_ as a signalling intermediate in TCR and co‐stimulatory signalling cascades, and the importance of PI3K modulation in both T‐cell exhaustion and leukaemic transformation, we hypothesised that INPP4B may serve as a critical, yet underappreciated, negative regulator of PI3K signalling in T cells.

In this study, we identified INPP4B, an inositol polyphosphate 4‐phosphatase of the PI3K pathway, as a T‐cell‐specific activation suppressor. Through multi‐omics integration analysis, we found that *INPP4B* forms a T‐cell‐specific chromatin interaction domain and is highly expressed in quiescent T cells. We revealed that the high expression of *INPP4B* in T cells is mediated by the T‐cell‐specific TF BCL11B. Additionally, we observed increased expression levels of *INPP4B* in exhausted T cells within the tumour microenvironment. Our findings suggest that *INPP4B* could be a promising target for enhancing the efficacy of T‐cell‐mediated immune responses against tumours.

## 2 RESULTS

### 2.1 *INPP4B* is downregulated in activated T cells

**FIGURE 1:**
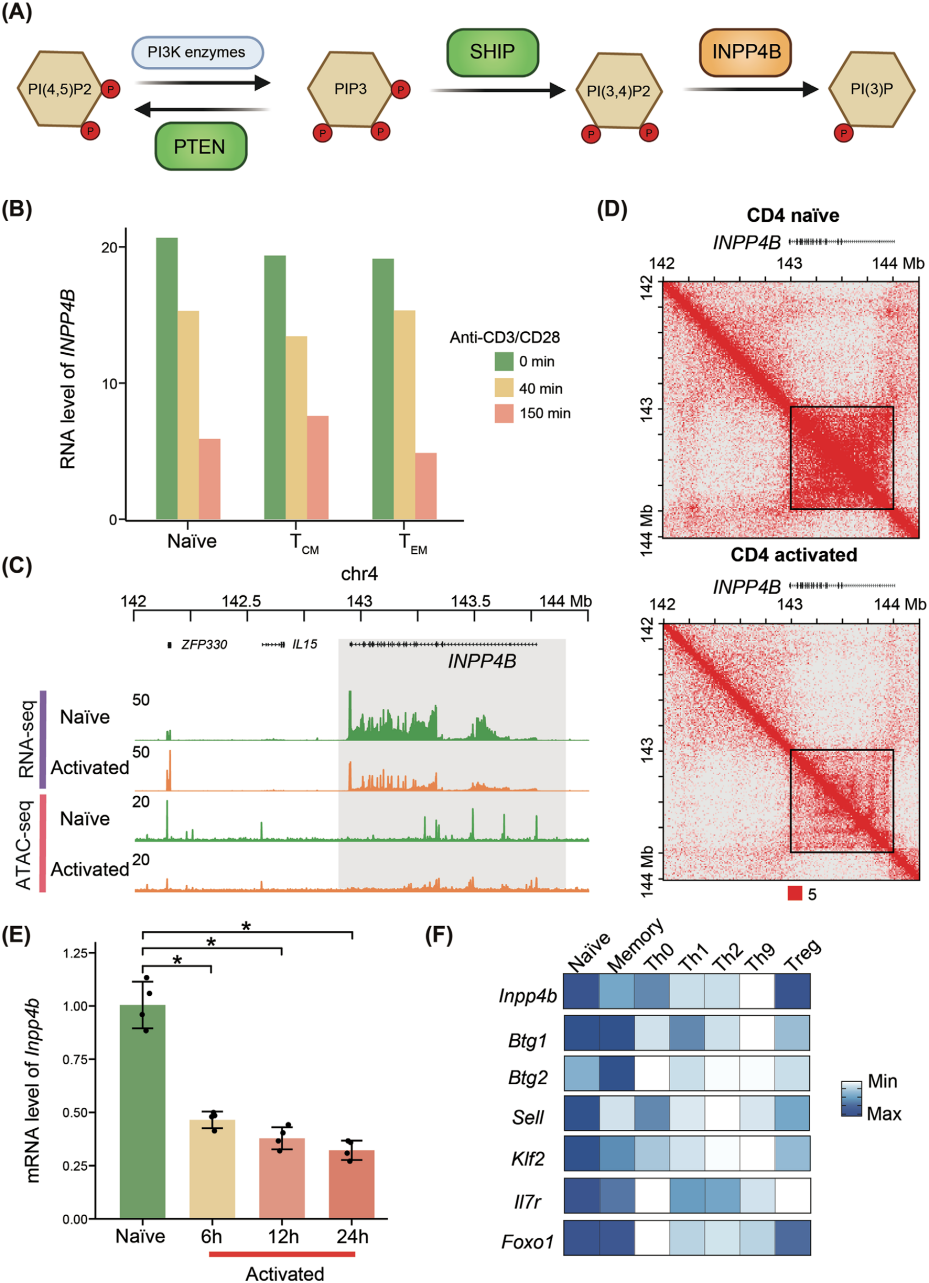
*INPP4B* is downregulated in activated T cells. (A) Schematic of phosphatases in phosphoinositide 3‐kinase (PI3K) pathway. (B) Expression level of *INPP4B* in naïve and memory CD4^+^ T cells upon activation. Naïve T cells, CD4^+^CD45RO^−^CD27^+^; central memory T cells, CD4^+^CD45RO^+^CD27^+^; effector memory T cells, CD4^+^CD45RO^+^CD27^−^. Re‐analysis results of published datasets.^15^ (C) Transcription level and chromatin accessibility around *INPP4B* in naïve CD4^+^ T cells upon activation. Re‐analysis results of published datasets.^17^ (D) Chromatin interactions around *INPP4B* in naïve CD4^+^ T cells upon activation. Re‐analysis results of published datasets.^17^ (E) Expression level of *Inpp4b* in mouse T cells upon activation (*n* = 4). *p*‐Value was calculated with two‐sided Wilcoxon test. ^*^ *p* < .05. (F) Relative expression level of *Inpp4b* in mouse CD4^+^ T subsets. Th, helper T cells; Treg, regulatory T cells. Re‐analysis results of published datasets.^21^

Since INPP4B is an inositol polyphosphate 4‐phosphatase of the PI3K pathway and converts PI(3,4)P_2_ to PI(3)P (Figure 1A), we hypothesised that INPP4B is involved in T‐cell activation. To test this, we compared the expression levels of *INPP4B* before and after T‐cell activation using published transcriptomic data.^15^ Our analysis revealed a gradual reduction in *INPP4B* expression levels in human naïve and memory CD4^+^ T cells following activation with CD3/CD28 antibodies coated on microbeads (Figures 1B and S1A). Additionally, analysis of a published liquid chromatography with tandem mass spectrometry (LC‒MS/MS) dataset profiling protein expression in resting and activated immune cells^16^ showed that the protein level of INPP4B was also reduced following T‐cell activation (Figure S1B). These findings suggest that INPP4B functions in quiescent T cells and is suppressed upon T‐cell activation. Further analysis of published ATAC‐seq (the assay for transposase‐accessible chromatin using sequencing) datasets of human naïve and activated CD4^+^ T cells^17^ revealed a significant reduction in chromatin accessibility around *INPP4B*, consistent with the observed decrease in transcription levels (Figure 1C). Recent studies have reported a positive correlation between gene expression and chromatin interactions.^18^, ^19^ Gene‐body‐associated domains (GADs) have been defined to describe interaction domain that precisely overlap with highly expressed gene bodies.^20^ Re‐analysis of published Hi‐C (high‐throughput chromosome conformation capture) datasets of human naïve and activated CD4^+^ T cells,^17^ using previously reported method to identify GADs,^20^ revealed that *INPP4B* formed a significant GAD in quiescent T cells. However, the interaction strength within the *INPP4B* GAD structure decreased following T‐cell activation (Figure 1D). Therefore, our multi‐omics comparative analysis indicates that INPP4B is involved in suppressing T‐cell activation and maintaining the quiescent state.

Next, we assessed the expression level of *Inpp4b* in naïve and activated mouse T cells. Quantitative PCR (qPCR) analysis revealed a reduction in *Inpp4b* expression in mouse T cells following activation by anti‐CD3/CD28 antibodies (Figure 1E). In addition, we confirmed that *Inpp4b* was predominantly expressed in naïve and memory T cells rather than in other effector T‐cell subsets with published RNA sequencing (RNA‐seq) dataset^21^ (Figure 1F), consistent with other factors known to suppress T‐cell activation, such as *Btg1* and *Foxo1*.^5^, ^22^ Furthermore, re‐analysis of multi‐omics datasets of mouse CD8^+^ T cells during acute lymphocytic choriomeningitis virus (LCMV) infection^23^ demonstrated a decrease in *Inpp4b* expression in two effector T‐cell subsets, short‐lived effector cells and memory precursor effector cells, but slightly recovered in memory T cells (Figure S1C). Concordantly, we observed a decrease in the H3K27ac signal, commonly used as a marker for active enhancers, around *Inpp4b* in effector T cells while recovered in memory T cells (Figure S1D). Given that both naïve and memory T cells are maintained in a quiescent state, these findings indicate that *Inpp4b* expression may be involved in suppressing T‐cell activation. Therefore, the above analyses demonstrate the strong association between INPP4B and T‐cell activation suppression is conserved in both human and mouse T cells.

### 2.2 *INPP4B* suppresses the activation of T cells

**FIGURE 2:**
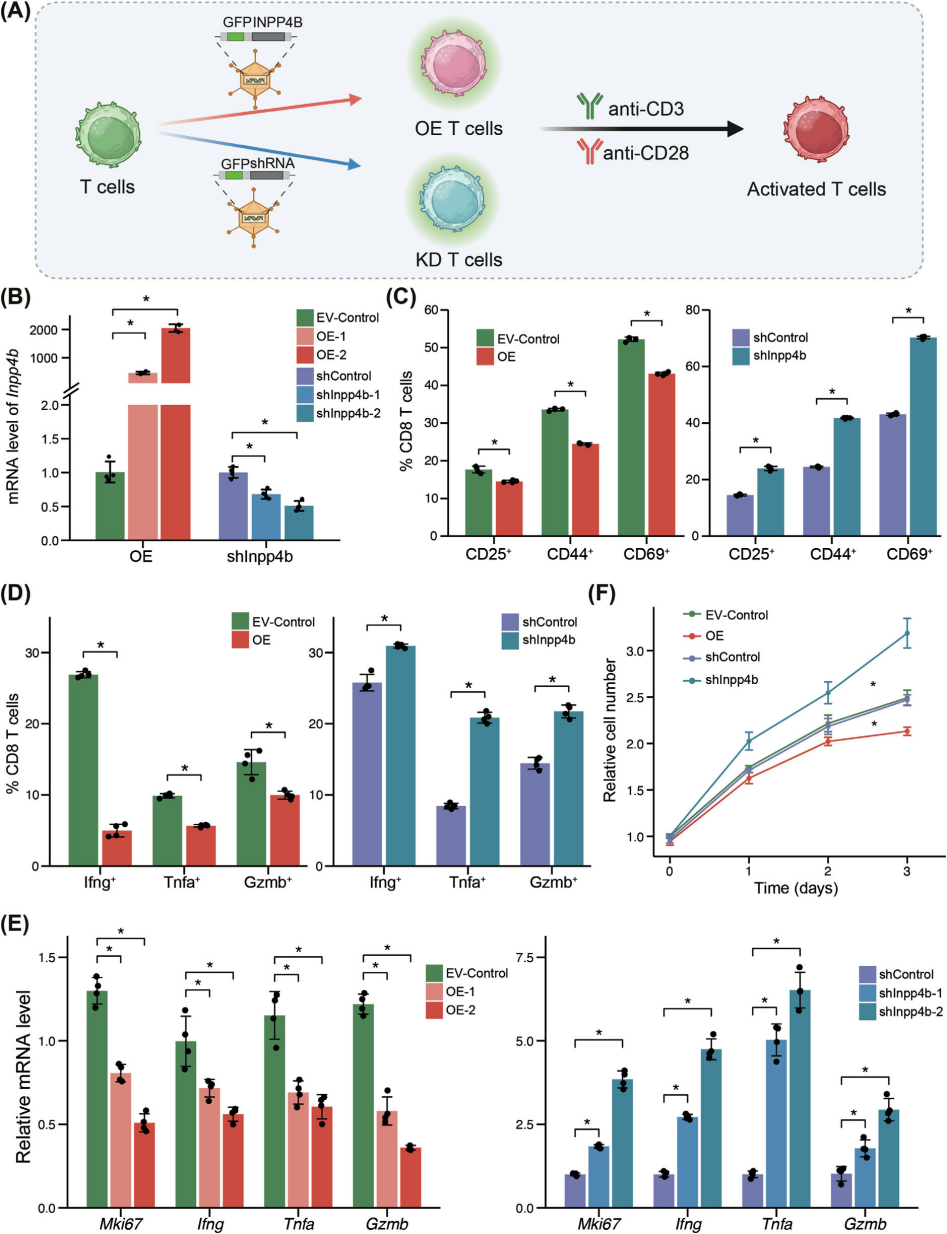
*INPP4B* suppresses T‐cell activation and effector functions. (A) Schematic of *Inpp4b* overexpression (OE) and knockdown (KD) in mouse T cells. (B) Expression level of *Inpp4b* in EV‐control (empty plasmid vector [EV] transfected cells), OE, shControl (non‐targeting shRNA control) and KD mouse CD8^+^ T cells (*n* = 4). (C) Percentage of CD25^+^, CD44^+^ and CD69^+^ subsets in anti‐CD3/CD28‐activated EV‐control, OE, shControl and KD mouse CD8^+^ T cells (*n* = 4). (D) Percentage of Ifng^+^, Tnfa^+^, Gzmb^+^ subsets in anti‐CD3/CD28‐activated EV‐control, OE, shControl and KD mouse CD8^+^ T cells (*n* = 4). (E) Expression level of *Mki67*, *Ifng*, *Tnf* and *Gzmb* in anti‐CD3/CD28‐activated EV‐control, OE, shControl and KD mouse CD8^+^ T cells (*n* = 4). (F) Accumulated proliferation of anti‐CD3/CD28‐activated EV‐control, OE, shControl and KD mouse CD8^+^ T cells. *p*‐Value was calculated with two‐sided Wilcoxon test. ^*^ *p* < .05.

To further explore the regulatory role of INPP4B in T‐cell activation, we designed short‐hairpin RNA (shRNA) to knockdown (KD) *Inpp4b* and constructed plasmids for overexpressing (OE) *Inpp4b* in primary mouse CD8^+^ T cells (Figure 2A). qPCR analysis confirmed successful KD and overexpression of *Inpp4b* (Figure 2B). Then, we stimulated these T cells with anti‐CD3/CD28 antibodies and assessed the frequencies of activated T‐cell subsets using flow cytometry with canonical activation markers CD25, CD44 and CD69.^24^ We observed significantly higher frequencies of CD25^+^, CD44^+^ and CD69^+^ cells within total CD8^+^ T cells in the KD group, and lower frequencies in the OE group (Figures 2C and S2A), demonstrating the suppression effect of INPP4B on T‐cell activation. Activated T cells can undergo further differentiation, giving rise to effector T cells that express cytokines such as Ifng, Tnfa and Gzmb.^24^ Similarly, we assessed the frequencies of effector subsets via flow cytometry and found that the KD group exhibited increased frequencies of Ifng^+^, Tnfa^+^ and Gzmb^+^ subsets, whereas the OE group showed decreased frequencies, indicating a negative regulatory role of INPP4B on effector T cells (Figures 2D and S2B). Furthermore, we evaluated the expression levels of proliferation marker *Mki67* and T‐cell cytokines (*Ifng*, *Tnf* and *Gzmb*). Compared to the control group, the KD group exhibited elevated expression levels of *Mki67* and cytokines, while the OE group showed decreased expression levels (Figure 2E). Additionally, we compared the T‐cell proliferation capacity among the three groups, finding reduced proliferative potential in the OE group and enhanced proliferative ability in the KD group (Figure 2F). Jurkat cells, an immortalised human T‐cell line, showed diminished expression of *INPP4B* compared to normal naïve T cells, accompanied by reduced chromatin interactions within the *INPP4B* GAD structure (Figures 1C,D and S2C,D). Subsequently, we overexpressed *INPP4B* in Jurkat cells (Figure S2E). Upon stimulation with CD3 and CD28 antibodies, significant reductions in the expression levels of *IFNG* and *TNF* were observed within the OE group (Figure S2F), further demonstrating the suppressive effect of INPP4B on the activation process of T cells. Therefore, these findings demonstrated the suppressive role of INPP4B in T‐cell activation and effector functions.

**FIGURE 3:**
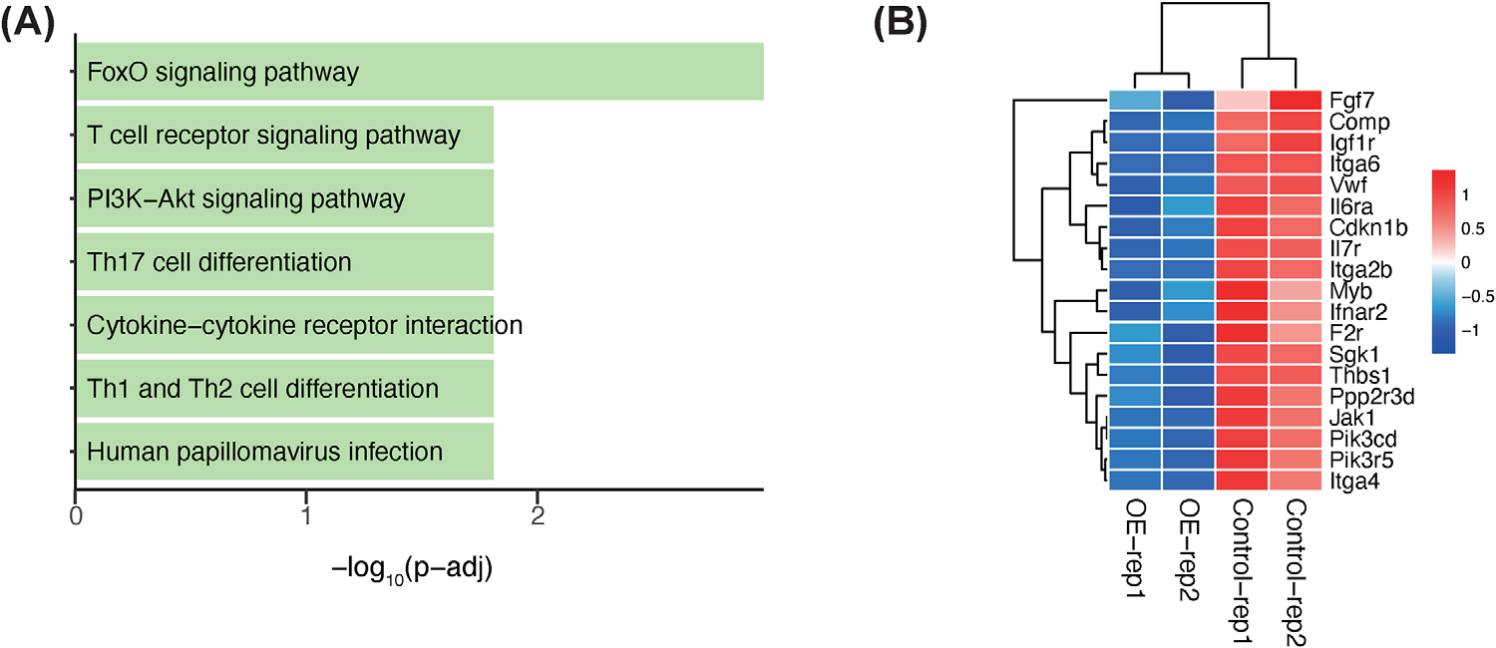
*INPP4B* overexpression suppresses phosphoinositide 3‐kinase (PI3K)‒protein kinase B (AKT) pathway. (A) Enriched pathways of downregulated genes in overexpression (OE) group. (B) Expression heatmap of representative downregulated genes in PI3K‒AKT pathways.

To investigate whether INPP4B regulates T‐cell activation through the PI3K‒AKT pathway, we stimulated control and *Inpp4b‐*overexpression mouse T cells with anti‐CD3/CD28 antibodies and performed RNA‐seq. Our analysis revealed significant enrichment of downregulated genes in the overexpression group within TCR, PI3K‒AKT and JAK‒STAT signalling pathways (Figure 3A,B). These results indicate that INPP4B inhibits T‐cell activation by attenuating PI3K‒AKT signalling.

### 2.3 *INPP4B* is actively expressed specifically in T cells

**FIGURE 4:**
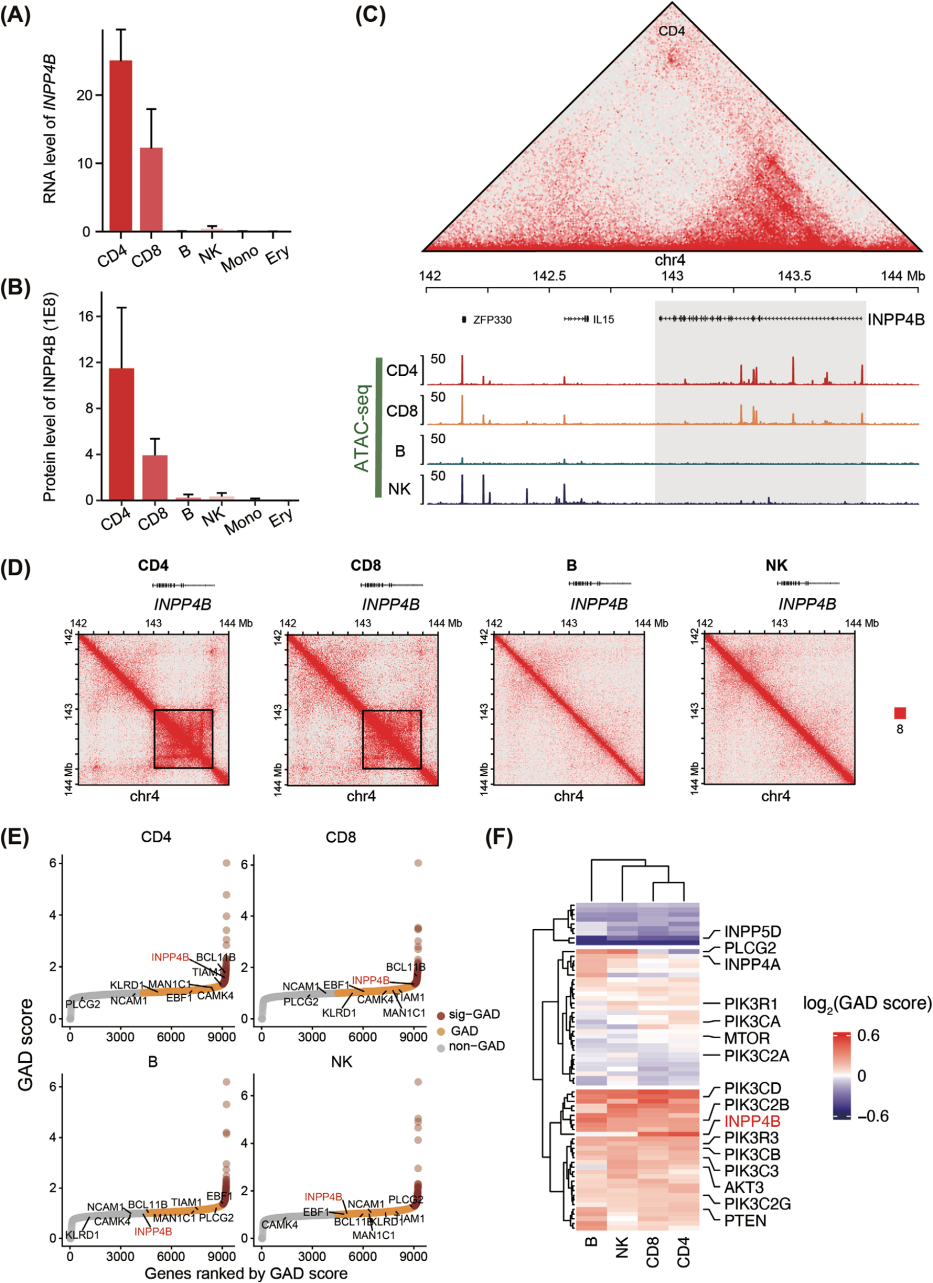
*INPP4B* is actively expressed in T cells. RNA (A) and protein (B) levels of *INPP4B* in human immune cells (*n* = 4). Re‐analysis results of published datasets.^16^, ^27^ (C) Chromatin accessibility around *INPP4B* in human immune cells. The top panel is the interaction heatmap around *INPP4B* gene‐body‐associated domain (GAD) structure in CD4^+^ T cells. (D) Chromatin interactions around *INPP4B* in human immune cells. (E) GAD score of all genes in human immune cells. Genes in phosphoinositide 3‐kinase (PI3K) pathway were marked out. (F) GAD score of members in PI3K pathway in human immune cells. (C‒F) Re‐analysis results of published datasets.^28^, ^29^, ^30^, ^31^

Next, we sought to determine whether INPP4B also functions in other immune cells. To this end, we analysed RNA‐seq, LC‒MS/MS, ATAC‐seq and Hi‐C datasets from human and mouse immune cells^16^, ^25^, ^26^, ^27^, ^28^, ^29^ to compare the expression levels and epigenome states of *INPP4B*. Our analysis revealed that both the RNA and protein levels of *INPP4B* were highest in T cells, while being limitedly expressed in other immune cell types, which was conserved in human and mouse (Figures 4A,B and S3A,B). Consistently, ATAC‐seq data indicated higher chromatin accessibility in the genomic regions around *INPP4B* in T cells compared to other immune cell types (Figures 4C and S3C). Additionally, we found that *INPP4B* specifically formed a significant GAD in T cells, but not in B or natural killer (NK) cells (Figures 4D and S3D). This finding correlates with the high expression level of *INPP4B* observed in T cells and is consistent with previous studies that reported a positive correlation between GADs and gene expression.^18^, ^30^ Moreover, among the members of the PI3K signalling pathway, only *INPP4B* exhibited T‐cell‐specific expression and epigenomic characteristics (Figure 4E,F). Therefore, these findings demonstrate that *INPP4B* forms a T‐cell‐specific GAD and is highly expressed in T cells, laying the foundation of its unique functions in T‐cell activities.

### 2.4 BCL11B regulates the transcription of *INPP4B* in T cell

**FIGURE 5:**
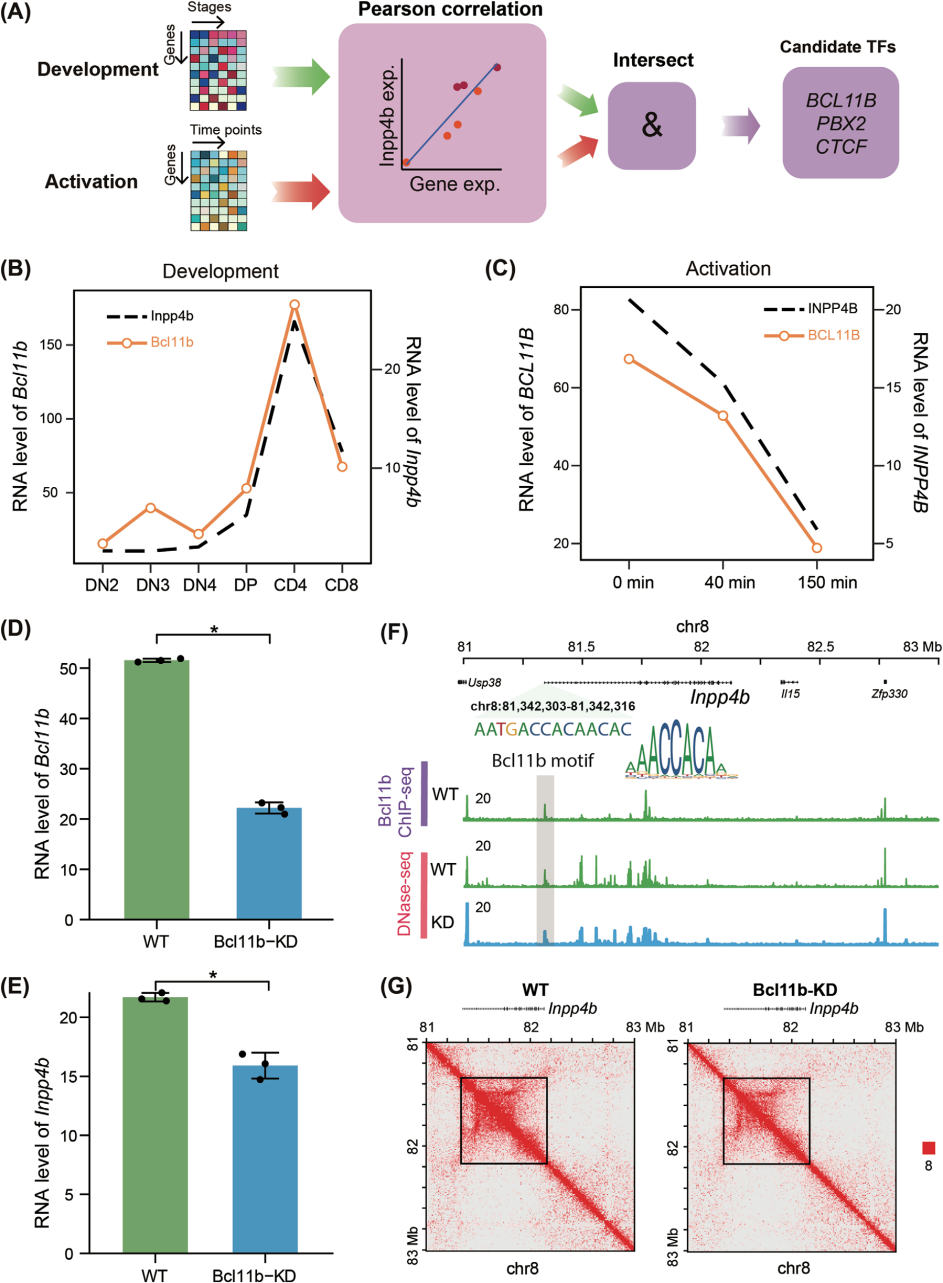
BCL11B regulates the transcription of *INPP4B*. (A) Schematic of analysis pipeline for screening transcription factors (TFs) whose expression levels positively correlated with *INPP4B*. Line plot of the expression levels of *Inpp4b* and *Bcl11b* during T‐cell development (B) and upon T‐cell activation (C). Re‐analysis results of published datasets.^15^, ^18^ Expression levels of *Bcl11b* (D) and *Inpp4b* (E) in the wildtype (WT) and *Bcl11b*‐knockdown (*Bcl11b*‐KD) naïve CD4^+^ T cells (*n* = 3). *p*‐Value was calculated with two‐sided Wilcoxon test. ^*^ *p* < .05. (F) Bcl11b binding signal and chromatin accessibility around *Inpp4b* in the WT and *Bcl11b*‐KD naïve CD4^+^ T cells. TSS, transcription start site. (G) Chromatin interaction heatmap around *Inpp4b* in the WT and *Bcl11b*‐KD naïve CD4^+^ T cells. (D‒G) Re‐analysis results of published datasets.^18^

Next, we explored the mechanisms underlying the transcriptional regulation of *INPP4B* in T cells. We hypothesised that specific TFs are selectively activated during T‐cell development, thereby orchestrating the distinctive elevation of *INPP4B* expression. Consequently, we examined the dynamics of *INPP4B* expression during T‐cell development (Figure S4A). The differentiation process from haematopoietic stem and progenitor cells to mature CD4 or CD8 single‐positive (SP) T cells involves several intermediate stages, including multipotent progenitor, common lymphoid progenitor, early T precursor, CD4 and CD8 double‐negative and double‐positive (DP) cells.^31^ Through the analysis of the transcription profile of mouse T cells at various developmental stages,^18^ we observed a significant upregulation of *Inpp4b* during the DP‐to‐SP transition (Figure S4B). Additionally, both the chromatin accessibility of *Inpp4b* and the interaction strength within the GAD structure were enhanced at this stage (Figure S4C,D). Based on these findings, we hypothesised that TFs activated during the DP‐to‐SP transition promote the transcription of *INPP4B*. We then conducted a computational screening to identify TFs upregulated during the DP‐to‐SP transition and downregulated upon T‐cell activation (Figure 5A). Three candidate TFs, including *BCL11B*, *CTCF*, *PBX2*, were identified (Figure S5A,B). Notably, *BCL11B* exhibited specific high expression in both human and mouse T cells (Figure S5C,H), with its expression positively correlated with *INPP4B* levels (Figure 5B,C). These results suggest that BCL11B may serve as a regulatory factor for the increased transcriptional levels of *INPP4B* in T cells.

To validate the regulatory role of BCL11B in *INPP4B* transcription, we analysed public multi‐omics datasets from *Bcl11b*‐KD mice.^18^ The analysis revealed a significant reduction in *Inpp4b* expression in *Bcl11b*‐KD naïve CD4^+^ T cells (Figure 5D,E). Furthermore, chromatin immunoprecipitation sequencing (ChIP‐seq) results validated the binding of Bcl11b to the promotor region of *Inpp4b* (Figure 5F,G). Following *Bcl11b*‐KD, both the chromatin accessibility level around *Inpp4b* and the interaction strength within the *Inpp4b* GAD structure decreased (Figure 5F,G). In conclusion, these results demonstrate the regulatory role of BCL11B in the transcription of *INPP4B*.

### 2.5 *INPP4B* was dysregulated in exhausted T cells and T‐cell leukaemia

**FIGURE 6:**
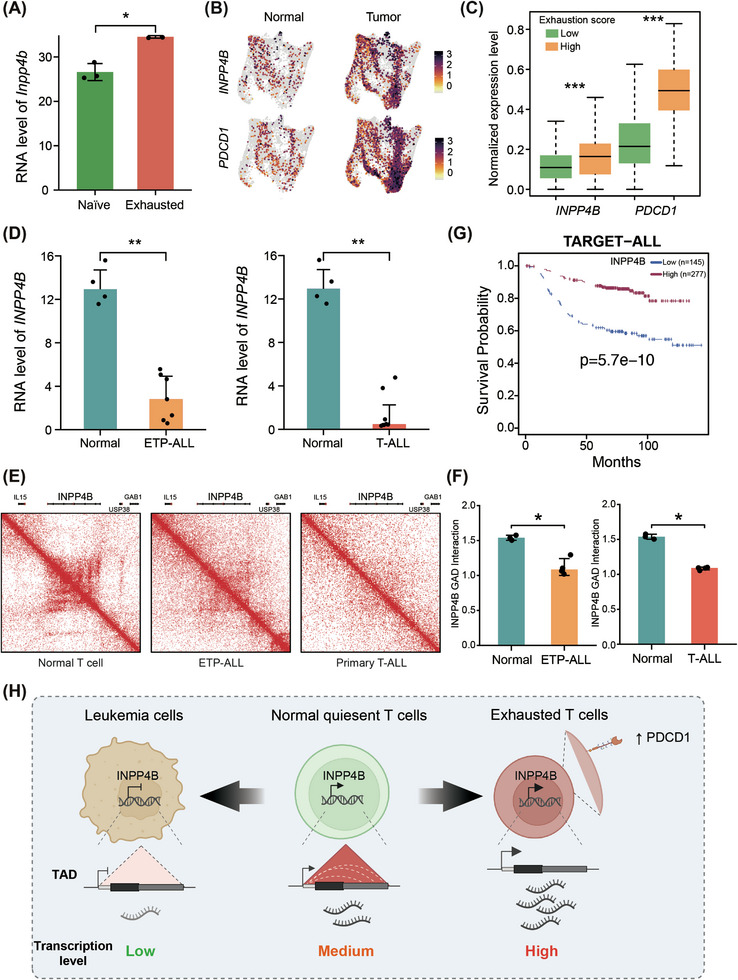
*INPP4B* is dysregulated in T cells under disease conditions. (A) Expression level of *Inpp4b* in naïve and exhausted OT‐I T cells isolated from mouse B78ChOVA melanomas. *p*‐Value was calculated with two‐sided Wilcoxon test. ^*^ *p* < .05. Re‐analysis results of published datasets.^33^ (B) UMAP projections of gene expression level for *INPP4B* and *PDCD1*. Re‐analysis results of published datasets.^34^ (C) Expression levels of *INPP4B* and *PDCD1* in exhausted T cells with higher (exhaustion score > 0) and lower (exhaustion score < 0) exhaustion score. *p*‐Value was calculated with two‐sided Wilcoxon test. ^***^ *p* < .001. Re‐analysis results of published datasets.^34^ (D) Expression levels of *INPP4B* in normal T cells, early T‐lineage progenitor acute lymphoblastic leukaemia (ETP‐ALL) and T‐cell acute lymphoblastic leukaemia (T‐ALL) cells. *p*‐Value was calculated with two‐sided Wilcoxon test. ^**^ *p* < .01. (E) Chromatin interactions around *INPP4B* in normal T cells, ETP‐ALL and T‐ALL cells. (F) *INPP4B* gene‐body‐associated domain (GAD) score in normal T cells, ETP‐ALL and T‐ALL cells. *p*‐Value was calculated with two‐sided Wilcoxon test. ^*^ *p* < .05. (D‒F) Re‐analysis results of published datasets.^38^ (G) Kaplan‒Meier overall survival curves for expression level of *INPP4B* in T‐ALL patients. (H) Schematic of expression levels of *INPP4B* in normal, exhausted and leukaemia T cells.

T cells can become exhausted, characterised by high expression of inhibitory receptors, poor effector function and decreased proliferative potential, when exposed to continuous stimulation under chronic infection or within the cancer microenvironment.^32^ Given that INPP4B suppresses T‐cell activation and effector functions, we hypothesised that dysregulation of *INPP4B* might be linked to T‐cell exhaustion. To test this, we re‐analysed the transcriptome dataset of OT‐I T cells, expressing a TCR that specifically recognises the ovalbumin peptide SIINFEKL, isolated from mouse B78ChOVA melanomas^33^ and observed increased expression of *Inpp4b* in exhausted T cells compared to naïve T cells (Figure 6A). In addition, we noted a concordant increase in chromatin accessibility around *Inpp4b* in exhausted T cells (Figure S6A). To further investigate whether *INPP4B* is upregulated in tumour‐infiltrating T cells, we re‐analysed a single‐cell RNA sequencing (scRNA‐seq) dataset of human lung cancer.^34^ T cells from tumour and normal tissue were clustered into nine clusters using canonical correlation analysis‐aligned methods (Figure S6B,C). Our analysis revealed that *INPP4B* exhibited upregulation in exhausted T cells, similar to exhaustion markers such as *PDCD1* (Figure 6B). Moreover, we computed an exhaustion score based on the expression of exhaustion marker genes (*PDCD1*, *TNFRSF9*, *LAG3*, *HAVCR2*, *TOX* and *CTLA4*), finding that T cells with higher exhaustion levels exhibited significantly higher *INPP4B* expression compared to less exhausted T cells (Figure 6C), indicating a strong correlation between *INPP4B* upregulation and T‐cell exhaustion. Pan‐cancer scRNA‐seq analysis of T cells from 316 patients across 21 cancer types^35^ further revealed that *INPP4B* is upregulated in exhausted T cells (Figure S6D), and its expression positively correlates with the exhaustion score computed based on the expression levels of exhaustion markers (Figure S6E,G). Therefore, these findings indicate that *INPP4B* overexpression is associated with the unresponsive state of exhausted T cells.

Given the diminished expression of *INPP4B* observed in Jurkat cells (Figure S2C), derived from a T‐ALL patient, we next investigated whether *INPP4B* dysregulation is common in T‐ALL patients. T‐ALL includes canonical T‐ALL, characterised by frequent *NOTCH1* mutations and an immature T‐cell phenotype, and early T‐lineage progenitor (ETP) leukaemia, characterised by frequently expressing stem cell and myeloid cell‐surface markers.^36^, ^37^ Comparative analysis revealed significant downregulation of *INPP4B* in both ETP‐ALL and T‐ALL patients compared to normal T cells (Figure 6D). Additionally, chromatin interactions were consistently reduced in both ETP‐ALL and T‐ALL patients (Figures 6E,F and S6H), suggesting that the transcriptional inhibition of *INPP4B* may contribute to the progression of T‐ALL. Furthermore, we found lower expression levels of *INPP4B* were significantly corrected with poor prognosis in T‐ALL patients (Figure 6G), indicating that *INPP4B* may serve as a therapeutic target for T‐ALL.

In summary, these findings suggest that maintaining INPP4B levels within a moderate range is crucial for T cells. Elevated levels are associated with an unresponsive and exhausted state, while diminished levels may contribute to the progression or leukaemia (Figure 6H).

## 3 DISCUSSION

T‐cell quiescence must be actively maintained by many regulators.^1^ Previous studies have highlighted the roles of co‐inhibitory immune receptors and TFs, such as BACH2 and FOXO1, in enforcing T‐cell quiescence.^39^ Recent investigations have uncovered additional regulators of T‐cell quiescence, including the inhibitory receptor VISTA expressed on the cell surface, as well as the regulation of RNA degradation and protein abundance by BTG1/2.^22^, ^40^ In this study, we reported that PI3K signalling member INPP4B is involved in suppressing T‐cell activation and maintaining quiescence. *INPP4B* exhibits a distinct expression pattern, with robust expression in naïve cells and repression in activated or effector cells, suggesting that silencing *INPP4B* transcription is crucial in T‐cell activation. Xu et al. reported that *Inpp4b* ^−/−^ mice did not show differences in T‐cell activation, proliferation and differentiation,^41^ indicating the presence of potential compensating factors for Inpp4b. INPP4A is a potential candidate, sharing 37% amino acid sequence identity and possessing a similar domain architecture with INPP4B.^42^

Exhausted T cells typically exhibit a progressive loss of effector functions, leading to decreased efficacy in clearing infected or malignant cells.^43^ Understanding T‐cell exhaustion is crucial in the fields of immunology and cancer immunotherapy, as strategies to reverse or prevent exhaustion can enhance the efficacy of T‐cell‐mediated immune responses against chronic infections or tumours.^44^ In this study, our functional experiments have confirmed that *INPP4B* overexpression suppresses T‐cell activation and effector functions. Furthermore, using pan‐cancer scRNA‐seq datasets of tumour‐infiltrating T cells, we revealed that *INPP4B* is upregulated in exhausted T cells within the tumour microenvironment. These findings suggest that INPP4B may function as a mediator of T‐cell exhaustion, contributing to the loss of effector functions in tumour‐infiltrating T cells. Our results indicate that INPP4B could be a potential target for preventing exhaustion and enhancing the efficacy of T‐cell‐mediated immune responses against tumours.

The PI3K signalling network has been reported to play a critical role in the development, proliferation, differentiation and activation of immune cells.^45^, ^46^, ^47^, ^48^ In this study, we uncovered that INPP4B, a phosphatase of the PI3K pathway, exhibits specific high expression in T cells while showing limited expression in other immune cell types. Our findings indicate that INPP4B may play a unique and indispensable role in maintaining T‐cell quiescence. AKT serves as a crucial downstream effector of PI3K signalling and it plays pivotal roles in the activation process of T cells.^49^ It is widely acknowledged that PI3K‒AKT signalling promotes the uptake of glucose and amino acids, as well as enhances protein synthesis in activated T cells.^50^ INPP4B, known for its ability to convert PI(3,4)P_2_ to PI(3)P and counteract PI3K‒AKT signalling,^51^ may regulate T‐cell quiescence by suppressing AKT signalling activation. Our RNA‐seq data revealed that genes downregulated in *Inpp4b* overexpression mouse T cells were enriched in the PI3K‒AKT pathway. Future studies should include functional validations to delve into mechanistic insights to comprehensively understand the regulatory mechanisms involved.

Our findings highlight the essential role of INPP4B in maintaining T‐cell quiescence and regulating activation thresholds through modulation of PI3K signalling. Given that PI3K signalling pathway has been implicated in the pathogenesis of several autoimmune disorders, including rheumatoid arthritis (RA) and MS, our study raises the possibility that INPP4B may also play a protective role in autoimmune contexts. In RA, inhibition of PI3K diminished neutrophil infiltration and Th17 differentiation.^52^ For MS, PI3Kγ and PI3Kδ isoforms are required for full development and persistence of autoimmune neuroinflammation in the experimental autoimmune encephalomyelitis (EAE) model, a widely used murine model of MS. In mice lacking functional PI3Kδ, T‐cell activation was markedly impaired, with reduced T‐cell infiltration into the central nervous system and increased apoptosis of T cells at early stages of EAE.^53^ These findings collectively highlight the essential role of PI3K signalling in supporting T‐cell survival, differentiation and pathogenicity during autoimmune responses. In this context, INPP4B may act as a brake on PI3K‐driven T‐cell activation and differentiation, suggesting that its modulation towards PI3K activity could influence T‐cell activity and autoimmune disease progression. Future studies are warranted to explore whether altered INPP4B expression or function contributes to the process of autoimmune diseases, and whether restoring INPP4B activity may represent a novel therapeutic strategy to prevent inappropriate T‐cell activation in autoimmune settings.

In summary, our study provided a transcriptome and epigenome profile of INPP4B in immune cells. We found that INPP4B is specifically highly expressed in T cells and is involved in suppressing T‐cell activation. Our results highlight the significance of maintaining *INPP4B* levels within a moderate range to ensure optimal T‐cell functions.

### 3.1 Study limitations

While our study provides compelling evidence that INPP4B is involved in modulating T‐cell activation and is preferentially expressed in quiescent T cells, we acknowledge several limitations. First, although we observed consistent transcriptional and functional changes in T cells upon INPP4B manipulation, our conclusions regarding its role in maintaining T‐cell quiescence are primarily based on in vitro assays and correlative analysis of multi‐omics datasets. Future work employing T‐cell‐specific, conditional INPP4B knockout mouse models will be necessary to fully resolve the role of INPP4B in T‐cell homeostasis and quiescence in vivo. Second, the absence of a specific antibody against phosphorylated INPP4B prevented us from evaluating its post‐translational regulation under different conditions. Given that INPP4B is a phosphoinositide phosphatase, its phosphorylation may influence its activity, localisation or interactions with signalling molecules. Future studies employing phospho‐specific detection methods or mass spectrometry‐based phosphoproteomics will be instrumental in further elucidating the regulatory mechanisms of INPP4B in T‐cell biology.

## 4 MATERIALS AND METHODS

### 4.1 Animals

Six‐ to eight‐week‐old C57BL/6 mice were purchased from Vital River Laboratory Animal Technology for CD8^+^ T‐cell isolation. All of the mice used in this study have been accredited by the Association for Assessment and Accreditation of Laboratory Animal Care International and Institutional Animal Care and Use Committee (IACUC) of Tsinghua University. All of the mice were maintained in pathogen‐free facilities and used strictly in accordance with the protocols approved by the IACUC of Tsinghua University. The study is compliant with all of the relevant ethical regulations regarding animal research. Sex was not considered in the study design. Only male mice were used for this study.

### 4.2 Cell lines

293T cells and Jurkat cells were gifts from the H. Wu laboratory (Peking University). 293T cells were cultured in DMEM medium (Macgene, CM10013) supplemented with 10% foetal bovine serum (FBS) and 100 U/mL penicillin/streptomycin. Jurkat cells were cultured in RMPI‐1640 medium (Macgene, CM10040) supplemented with 10% FBS and 100 U/mL penicillin/streptomycin. Primary mouse CD8^+^ T cells were cultured in RMPI‐1640 medium (Macgene, CM10040) containing 10% FBS, 100 U/mL penicillin/streptomycin and 10 ng/mL interleukin‐2. Primary CD8^+^ T cells were stimulated with anti‐CD3 (5 µg/mL, Biolegend, 100359) and anti‐CD28 (1 µg/mL, Biolegend, 102112) antibodies. All cell lines were cultured for no more than 2 months and have been authenticated and were tested for mycoplasma contamination.

### 4.3 DNA constructs and lentivirus production

Human INPP4B plasmid was constructed by cloning its cDNA into the pCHN1 vector. An shRNA fragment targeting the CDS of mouse Inpp4b was generated using a pair of primers (forward primer: 5′‐CCGGGCAGTCGGCATTTCAGATTTGCTCGAGCAAATCTGAAATGCCGACTGCTTTTTG‐3′ where the target sequence is underlined; reverse primer: 5′‐ AATTCAAAAAGCAGTCGGCATTTCAGATTTGCTCGAGCAAATCTGAAATGCCGACTGC ‐3′), and cloned into the plasmid pLKO.1‐TRC (Addgene 10878) as described in the TRC protocols (http://www.broadinstitute.org).

Lentivirus was produced in HEK293T cells by co‐transfecting the above plasmids together with lentivirus‐packaging vectors pMD2.G (Addgene 12259) and psPAX2 (Addgene 12260). Forty‐eight and 72 h after transfection, the culture supernatants containing the released viral particles were collected and, if needed, concentrated by ultracentrifugation of 20 000 rpm for 2.5 h.

### 4.4 Isolation and stimulation of mouse CD8+ T cells

Mouse T cells were isolated from the spleens of the mice using a biotin selection kit (Stem cell, 17665). The CD8^+^ T cells were, unless otherwise indicated, stimulated with 5 µg/mL anti‐CD3 (Biolegend, 100359) and 1 µg/mL anti‐CD28 (Biolegend, 102112) antibodies.

### 4.5 Stimulation of Jurkat cells

Jurkat cells were simulated with 5 µg/mL anti‐CD3 (Biolegend, 300465) and 1 µg/mL anti‐CD28 (Biolegend, 302943) antibodies for 6 h.

### 4.6 Lentiviral transduction

Primary CD8^+^ T cells were centrifugated and re‐suspended by corresponding media added with lentivirus and polybrene (Sigma AL‐118) at a final concentration of 8 µg/mL. After 6–10 h, the media were replaced with fresh viral‐free medium to allow further growth until use.

### 4.7 Reverse transcription and PCR analysis

Total RNA and cDNA were prepared using FastPure Cell/Tissue Total RNA Isolation Kit V2 (Vazyme RC112‐01) and HiScript III RT SuperMix for qPCR (+gDNA wiper) (Vazyme R323‐01). PCR was performed according to the manufacturer's instructions (Vazyme Q311‐02). The primer pairs for quantitative real‐time PCR are listed in Table S1.

### 4.8 Flow cytometry

All reagents and antibodies (anti‐CD69‐Pacific Blue, 104524; anti‐CD25‐APC, 102011; anti‐CD44‐PE, 103008; anti‐TNF‐α‐PE, 506306; anti‐IFN‐γ‐APC, 505810; anti‐Granzyme B‐PE/Cyanine7, 372213) for flow cytometric analysis were purchased from Biolegend. Flow cytometry was performed using a BD LSRFortessa SORP flow cytometer. For surface staining of CD8^+^ T cells to measure CD69, CD25 and CD44, cells were harvested and washed with phosphate‐buffered saline (PBS) for two times, then stained in PBS containing 2% FBS with antibodies at the recommended dilution on ice for 30 min in the dark and then washed twice with PBS prior to flow cytometry analysis. For intracellular of mouse primary CD8^+^ T cells to measure cytokines, cells were harvested and washed two times with PBS, fixed, permeabilised and stained with Intracellular Fixation & Permeabilisation Buffer set (eBioscience, 88‐8824) according to the manufacturer's protocol. Stained cells were washed two times with PBS prior to FACS analysis. FACS Diva software (BD) version 8.0 was used for FACS data collection. Positive events were determined by isotype control gating for each antibody. Cells were first gated using forward scatter (FSC)/side scatter (SSC) characteristics to exclude debris, followed by gating FSC‐A and FSC‐H, then SSC‐A and SSC‐H to eliminate nonsinglets. Then, target cells were gated the population of interest by specific stain. Data analysis was carried out using FlowJo (v10.0.7, FlowJo).

### 4.9 RNA‐seq analysis

Raw sequencing reads were download from the Gene Expression Omnibus (GEO) database and aligned to the human genome assembly (hg19) or mouse genome assembly (mm10) with STAR v2.7.2b.^54^ Then, gene expression levels were evaluated using the python package HTSeq^55^ with parameter ‘htseq‐count ‐q ‐f bam ‐r name ‐s no’. The downstream differential expression gene analysis was done with R package DESeq2^56^ and DEGs were defined with abs(log2 FoldChange) > 1 and *p* < .05. Gene set enrichment analysis were performed using Metascape.^57^

### 4.10 Hi‐C analysis

All raw sequencing reads were download from the GEO database and processed into .allvalidpairs files using the Hi‐C‐Pro pipeline.^58^ Then, the .allvalidpairs files were further transformed to .hic files with Juicer tools^59^ and visualised with Juicebox.^60^

### 4.11 ATAC‐seq and ChIP‐seq analysis

Raw sequencing reads were download from the GEO database and aligned to the human genome assembly (hg19) or mouse genome assembly (mm10) with Bowtie2 v2.3.5. The alignment parameters of ATAC‐seq data were ‘‐very‐sensitive ‐no‐mixed ‐no‐discordant ‐X 2000’. The alignment parameters of ChIP‐seq data were ‘‐local ‐very‐sensitive‐local ‐no‐unal ‐no‐mixed ‐no‐discordant ‐I 10 ‐X 700’.

### 4.12 GAD score analysis

GADs are defined based on ICE‐normalised matrix generated from HiC‐Pro output at 10 kb resolution.^20^ Generally, for each gene longer than 30 kb (≥3 bins), the average interactions intensity within gene body region (*G*) were compared to the upstream (*G*_u_) and downstream (*G*_d_) region in the range of equal gene length, respectively. The GAD scores (*G*_s_) were calculated as follows:

$$G_{s}=\frac{G^{2}}{G_{u}+G_{d}}$$

Genes were ranked and sorted based on GAD score. *G*_s_ of true GADs were supposed to be greater than 1. Significate gene‐associated domains (sig‐GAD) were defined as *G*_s_ > 1 and past the point where the slope is 1 (slope > 1).

### 4.13 Screening of candidate transcription factors regulating INPP4B

To identify TFs that regulate INPP4B expression, we performed Pearson correlation analyses between the expression levels of INPP4B and TFs using RNA‐seq datasets from T‐cell development^18^ and from T‐cell activation.^15^ TFs with a correlation coefficient >.3 and a *p* < .05 were considered significant. We then intersected the results from the two datasets to identify potential transcriptional regulators of INPP4B. Among them, BCL11B showed the highest correlation with INPP4B expression and exhibited T‐cell‐specific expression.

### 4.14 scRNA‐seq analysis

Reads count matrices were download from the GEO database and processed with the Seurat R package (version 4.1.1).^61^ Low‐quality cells with fewer than 400 genes, fewer than 1000 unique molecular identifiers or more than 10% of read counts corresponding to mitochondrial genes were filtered. Then, count data were log‐normalised and scaled to 10 000. Principal component analysis (PCA) was performed with the top 2000 most variable genes. The top 30 dimensions resulted from PCA analysis were used for correcting batch effect among different samples with Harmony (version 1.1.0).^62^ Finally, we calculated a UMAP for data visualisation with the top 30 dimensions resulted from PCA analysis and calculated clusters using the *k*‐nearest neighbors method with resolution parameter set to 1. Exhaustion scores were calculated using the ‘AddModuleScore’ function in Seurat.

### 4.15 Statistics

All statistical analyses were performed with R software. All statistical comparisons were performed only between two groups at a time using two‐sided Wilcoxon test. All data are presented as mean ± standard deviation.

### 4.16 Data visualisation

Hi‐C chromatin interaction heatmaps were visualised using Juicebox.^60^ Aligned bam files of RNA‐seq, ATAC‐seq and ChIP‐seq datasets were transformed to bigwig file with deeptools^63^ and visualised by pyGenomeTracks.^64^

## AUTHOR CONTRIBUTIONS

Ting Peng designed the study with supervision by Cheng Li. Ting Peng and Qing Fang performed data analysis. Ting Peng, Qing Fang, and Zihao Zhao performed the validation experiments. Ting Peng, Qing Fang, and Zihao Zhao drafted the manuscript. All the authors read and approved the final version.

## CONFLICT OF INTEREST STATEMENT

The authors declare they have no conflicts of interest.

## ETHICS STATEMENT

The study was approved by the Ethics Committee of Peking University.

## CONSENT FOR PUBLICATION

All the authors have read and approved the submission of the final manuscript.

## Supporting information



Supporting information

## Data Availability

Multi‐omics datasets of human and mouse immune cells were downloaded from GEO with accession numbers GSE75384, GSE60103 and GSE105918. Multi‐omics datasets of mouse T‐cell development were downloaded from GEO with accession number GSE79422. Multi‐omics datasets of T‐cell activation were downloaded from GEO with accession numbers GSE89404, GSE126117, GSE153862, GSE85951 and GSE150442. Processed LC‒MS/MS count file of activated T cells was provided by Rieckmann et al.^16^ Multi‐omics datasets of *Bcl11b*‐KD mice were downloaded from GEO with accession numbers GSE79422 and GSE223334.
