# Supplementary material for: Multi‐omics integration analysis identifies INPP4B as a T‐cell‐specific activation suppressor
Source: Clin Transl Med. 2025 Aug 3;15(8):e70430. doi: 10.1002/ctm2.70430 (PMC12318829; doi:10.1002/ctm2.70430)
Supplement: Supplementary file 1 — Supporting information [file CTM2-15-e70430-s001.docx]

**Supplemental information**

**
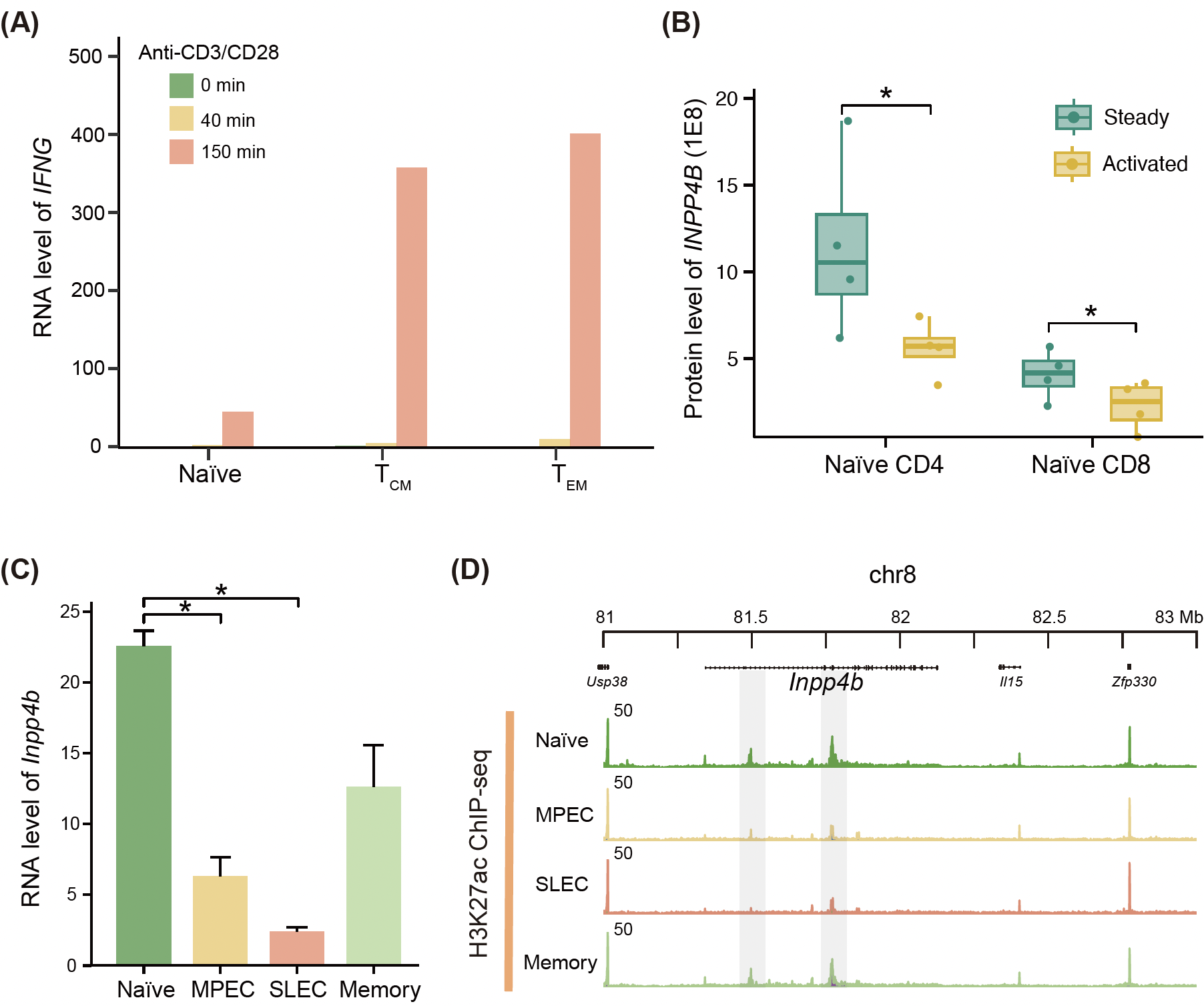
**

**Figure S1.** **(A)** RNA level of *IFNG* in naïve and memory CD4^+^ T cells upon activation. Re-analysis results of published datasets^1^. **(B)** Protein level of *INPP4B* in CD4^+^ and CD8^+^ T cells upon activation. p-value was calculated with two-sided Wilcoxon test. *, p-value<0.05. Re-analysis results of published datasets^2^. **(C)** Expression level of *Inpp4b* in mouse CD8^+^ T cell subsets (n = 3). p-value was calculated with two-sided Wilcoxon test. *, p-value<0.05. **(D)** H3K27ac signal around *Inpp4b* in mouse CD8^+^ T cell subsets. Figure S1C-D were re-analysis results of published datasets^3^.


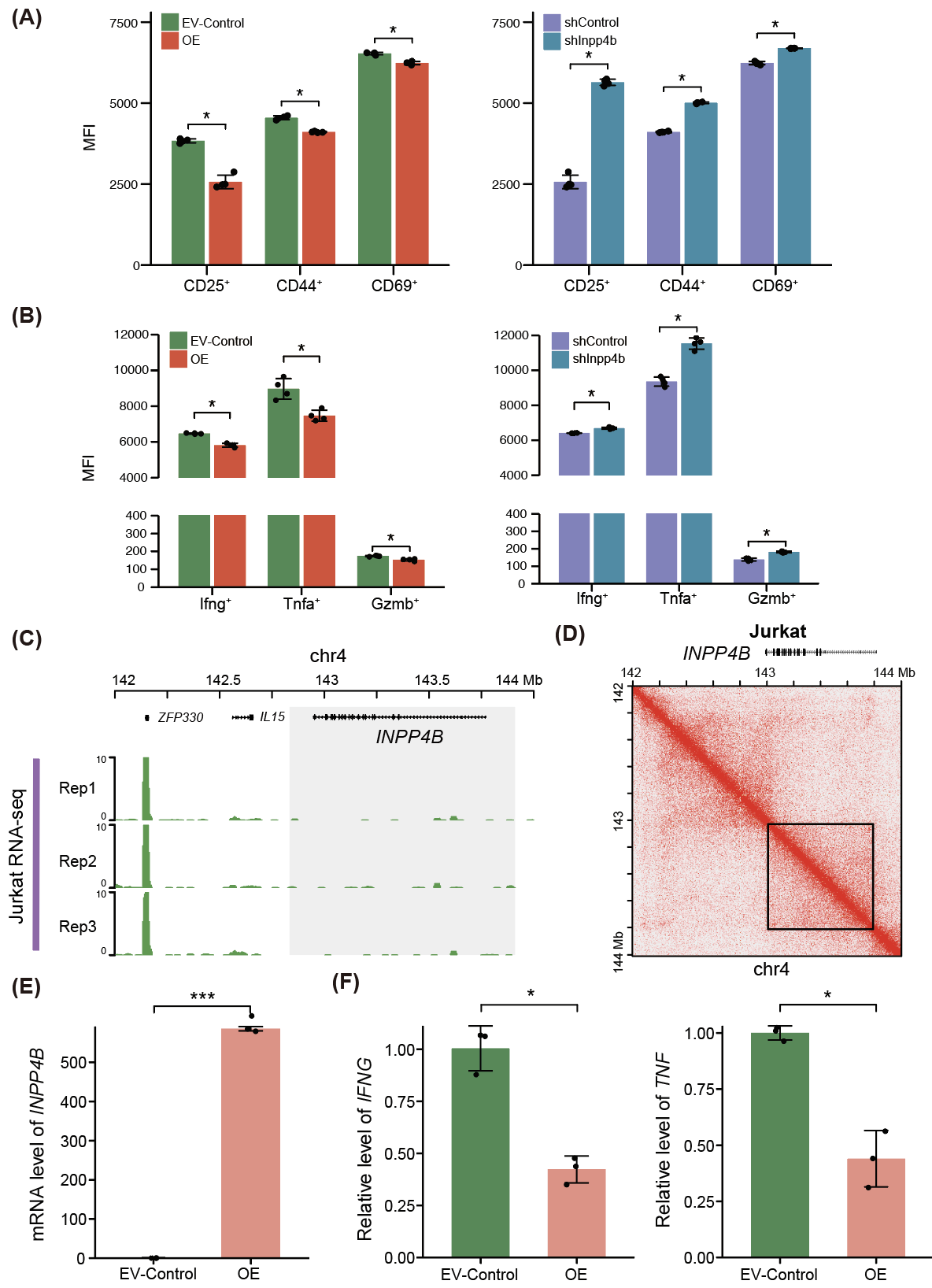


**Figure S2.** **(A)** Mean fluorescence intensity (MFI) of CD25, CD44, and CD69 in anti-CD3/CD28-activated mouse CD8^+^ T cells (n = 4). **(B)** MFI of Ifng, Tnfa, and Gzmb in anti-CD3/CD28-activated mouse CD8^+^ T cells (n = 4). **(C)** Expression level of *INPP4B* in Jurkat cells. **(D)** Chromatin interactions around *INPP4B* in Jurkat cells. **(E)** Expression level of *INPP4B* in control (empty plasmid vector) and OE Jurkat cells (n = 3). **(F)** Expression level of *IFNG* and *TNF* in anti-CD3/CD28-activated EV-Control and OE Jurkat cells (n = 3). p-value was calculated with two-sided Wilcoxon test. ***, p-value<0.001; *, p-value<0.05.


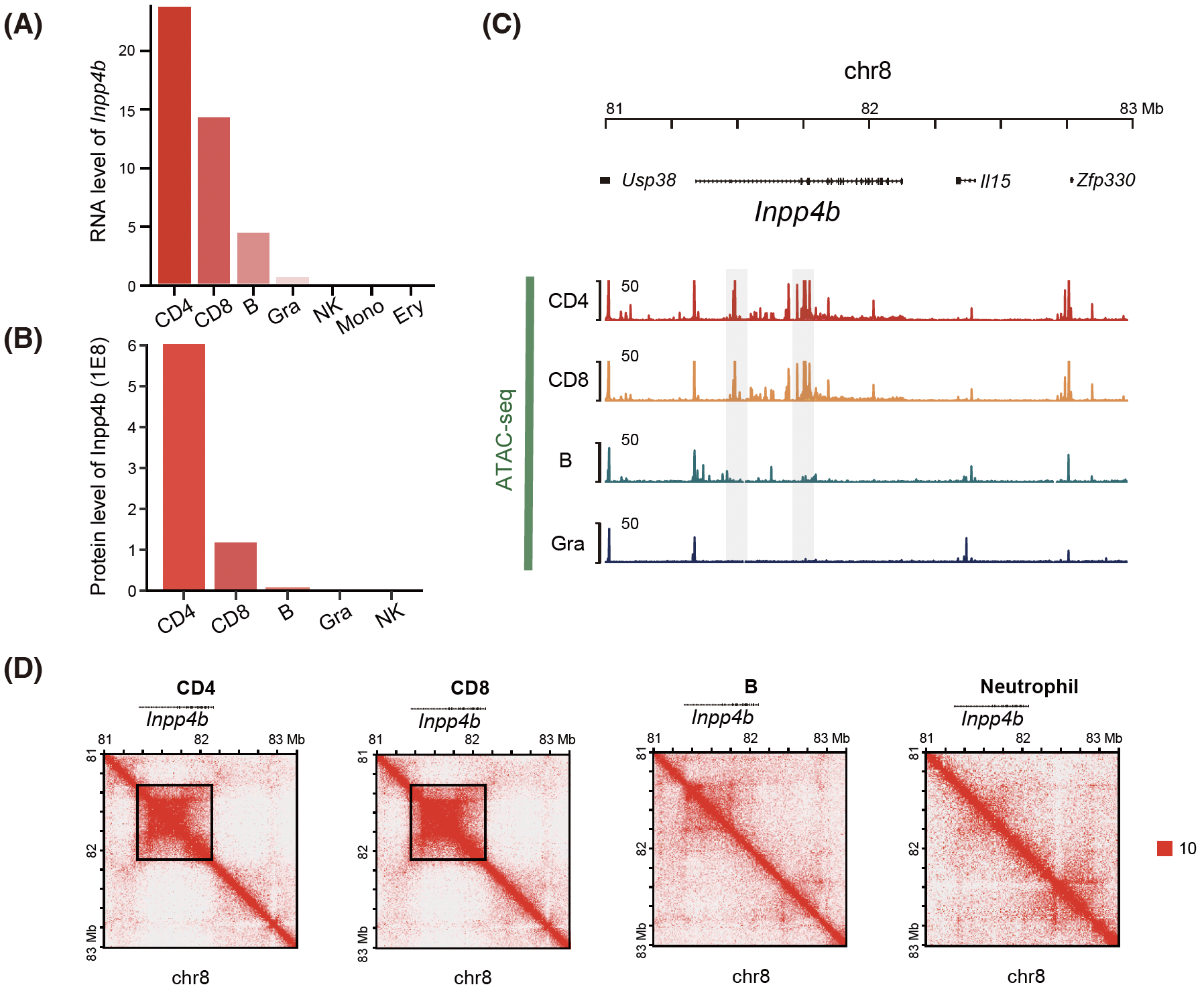


**Figure S3. (A-B)** RNA (A) and protein (B) levels of *Inpp4b* in mouse immune cells. **(C)** Chromatin accessibility around *Inpp4b* in mouse immune cells. **(D)** Chromatin interactions around *Inpp4b* in mouse immune cells. Figure S3A-D were re-analysis results of published datasets^4, 5, 6^.


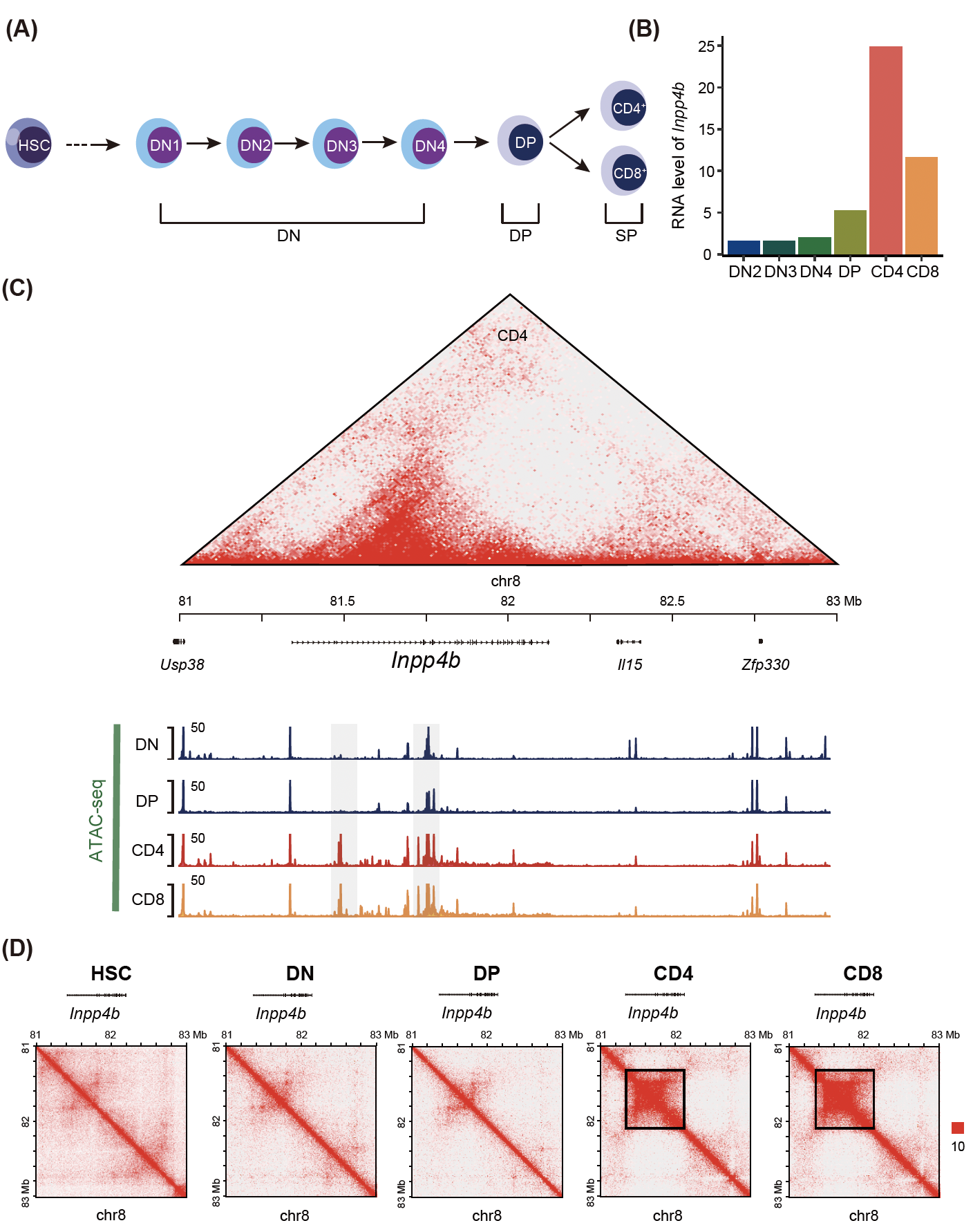


**Figure S4. (A)** Schematic of T cell development procedure. **(B)** Expression level of *Inpp4b* in DN, DP, and SP T cells. **(C)** Chromatin accessibility around *Inpp4b* in DN, DP, and SP T cells. The top panel is the interaction heatmap around *Inpp4b* GAD structure in CD4^+^ T cells. **(D)** Chromatin interactions around *Inpp4b* in HSC, DN, DP, and SP T cells. Figure S4B-D were re-analysis results of published datasets^4^.


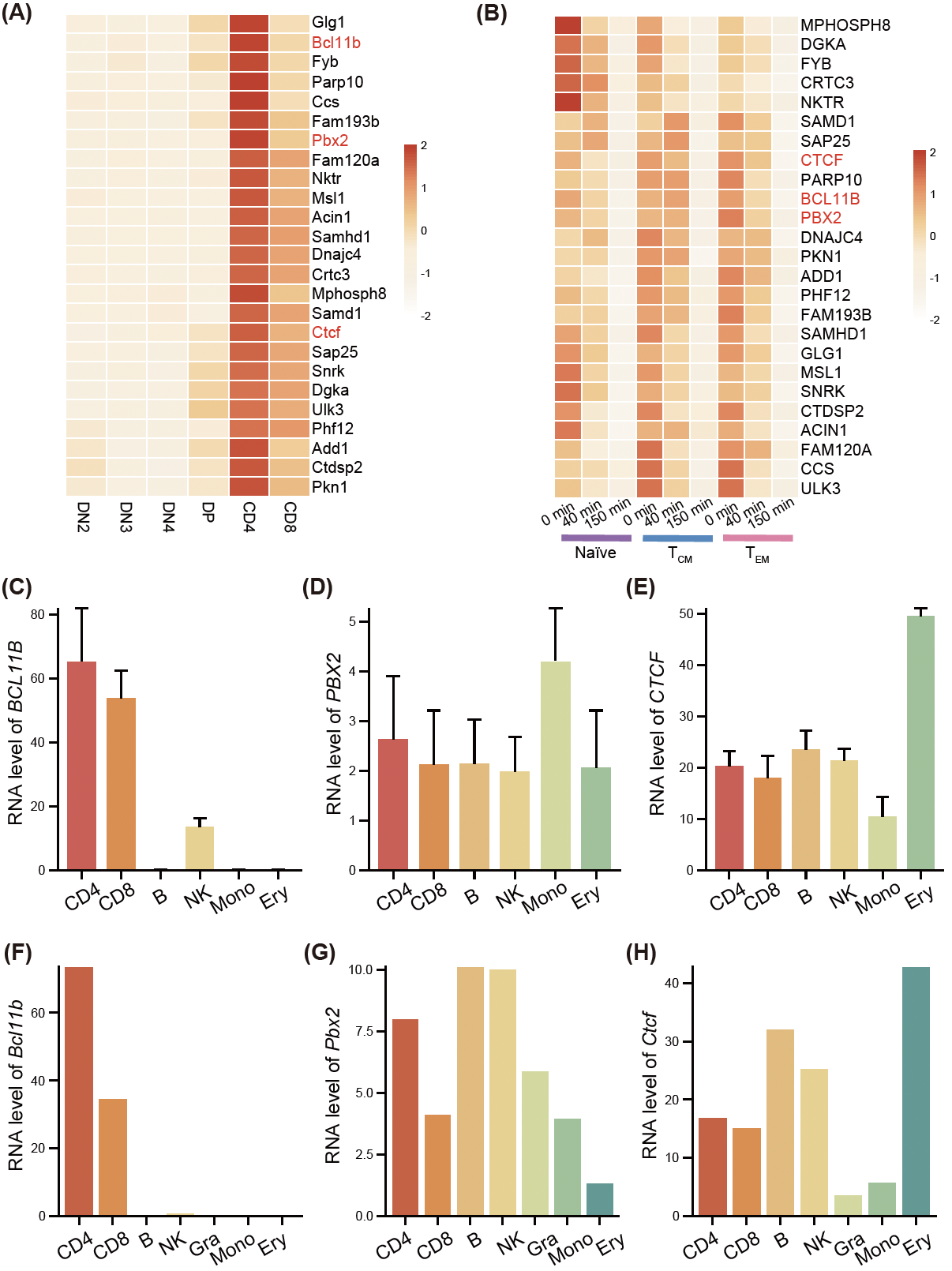


**Figure S5.** **(A-B)** Expression levels of the gene set whose expression levels positively correlated with *INPP4B* during T cell development (A) and upon T cell activation (B). Re-analysis results of published datasets^1, 4^. **(C-E)** Expression levels of *BCL11B* (C), *PBX2* (D), and *CTCF* (E) in human immune cells (n = 4). Re-analysis results of published datasets^7^. **(F-H)** Expression levels of *Bcl11b* (F), *Pbx2* (G), and *Ctcf* (H) in mouse immune cells. Re-analysis results of published datasets^5^.

**
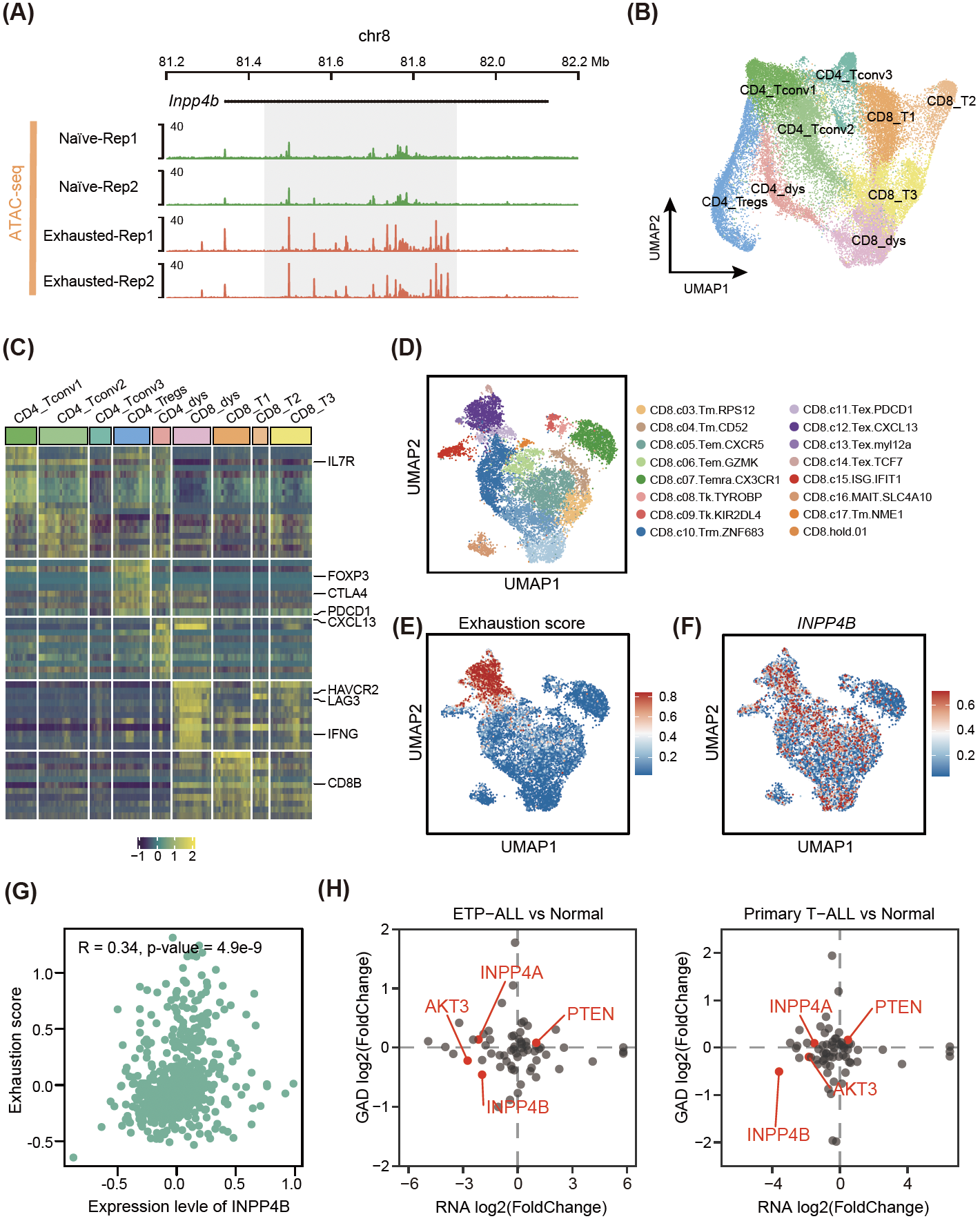
**

**Figure S6*.* (A)** Chromatin accessibility around *Inpp4b* in naïve and exhausted OT-I T cells isolated from mouse B78ChOVA melanomas. Re-analysis results of published datasets^8^. **(B)** UMAP projection of T cells from tumor and normal lung tissues. **(C)** Expression level of the marker genes of T cell subsets. Figure S6B-C were re-analysis results of published datasets^9^. **(D)** UMAP projections of pan-cancer tumor-infiltrating T cells. **(E-F)** UMAP projections of pan-cancer tumor-infiltrating T cells, dot colors were scaled by the exhaustion score (E) and expression level of *INPP4B* (F). **(G)** Correlation of the expression level of *INPP4B* and exhaustion score in pan-cancer tumor-infiltrating T cells. Correlation coefficient (R) and p-value were calculated by Pearson method. Figure S6D-G were re-analysis results of published datasets^10^. **(H)** GAD score and expression level changes of PI3K members in ETP-ALL and T-ALL patients. Re-analysis results of published datasets^11^.

**Table S1** qPCR primers

| **Gene** | **Forward** | **Reverse** |
| --- | --- | --- |
| Mouse *Inpp4b* | AGAACCTCAGATGGTGGCAAA | CCCGCTCAGACTTTCTGGTG |
| Mouse *Gzmb* | CCACTCTCGACCCTACATGG | GGCCCCCAAAGTGACATTTATT |
| Mouse *Tnfa* | CCCTCACACTCAGATCATCTTCT | GCTACGACGTGGGCTACAG |
| Mouse *Ifng* | ATGAACGCTACACACTGCATC | CCATCCTTTTGCCAGTTCCTC |
| Mouse *Actb* | GGCTGTATTCCCCTCCATCG | CCAGTTGGTAACAATGCCATGT |
| Mouse *Mki67* | ATCATTGACCGCTCCTTTAGGT | GCTCGCCTTGATGGTTCCT |
| Human *INPP4B* | CCAGAAGACTCCAAATGAACCG | ACGGGGTGGATTACGGAGA |
| Mouse *Inpp4b* | AGAACCTCAGATGGTGGCAAA | TCGCTTCCCTGTTTTAGCTGC |

References

1. Barski, A. *et al.* Rapid Recall Ability of Memory T cells is Encoded in their Epigenome. *Sci Rep-Uk* **7** (2017).

2. Rieckmann, J.C. *et al.* Social network architecture of human immune cells unveiled by quantitative proteomics. *Nat Immunol* **18**, 583-593 (2017).

3. Mitchell, J.E. *et al.* UTX promotes CD8(+) T cell-mediated antiviral defenses but reduces T cell durability. *Cell Rep* **35**, 108966 (2021).

4. Hu, G. *et al.* Transformation of Accessible Chromatin and 3D Nucleome Underlies Lineage Commitment of Early T Cells. *Immunity* **48**, 227-242 e228 (2018).

5. Lara-Astiaso, D. *et al.* Chromatin state dynamics during blood formation. *Science* **345**, 943-949 (2014).

6. Johanson, T.M. *et al.* Genome-wide analysis reveals no evidence of trans chromosomal regulation of mammalian immune development. *Plos Genetics* **14** (2018).

7. Corces, M.R. *et al.* Lineage-specific and single-cell chromatin accessibility charts human hematopoiesis and leukemia evolution. *Nature Genetics* **48**, 1193-1203 (2016).

8. Kersten, K. *et al.* Spatiotemporal co-dependency between macrophages and exhausted CD8 T cells in cancer. *Cancer Cell* **40**, 624-638 (2022).

9. Qian, J. *et al.* A pan-cancer blueprint of the heterogeneous tumor microenvironment revealed by single-cell profiling. *Cell Res* **30**, 745-762 (2020).

10. Zheng, L. *et al.* Pan-cancer single-cell landscape of tumor-infiltrating T cells. *Science* **374**, abe6474 (2021).

11. Kloetgen, A. *et al.* Three-dimensional chromatin landscapes in T cell acute lymphoblastic leukemia. *Nature Genetics* **52**, 388-+ (2020).
